# Do Online Trolling Strategies Differ in Political and Interest Forums: Early Results

**DOI:** 10.1007/978-3-030-61841-4_13

**Published:** 2020-10-19

**Authors:** Henna Paakki, Antti Salovaara, Heidi Vepsäläinen

**Affiliations:** 8 Leiden Institute of Advanced Computer Science, Leiden University, Leiden, The Netherlands; 9 Leiden Institute of Advanced Computer Science, Leiden University, Leiden, The Netherlands; 10 School of Politics and International Studies, University of Leeds, Leeds, UK; 11 Leiden Institute of Advanced Computer Science, Leiden University, Leiden, The Netherlands; 12 Leiden Institute of Advanced Computer Science, Leiden University, Leiden, The Netherlands; 13 Department of Computer Science, Aalto University, Espoo, Finland; 14 Department of Design, Aalto University, Espoo, Finland; 15 Department of Computer Science, University of Helsinki, Helsinki, Finland

**Keywords:** Trolling strategies, Political forum, Interest forum, Relevance theory, Common grounding.

## Abstract

This study compares the effectiveness of different trolling strategies in two online contexts: politically oriented forums that address issues like global warming, and interest-based forums that deal with people’s personal interests. Based on previous research, we consider trolling as context-bound and suggest that relevance theory and common grounding theory can explain why people may attend and react to certain types of troll posts in one forum, but pay scant attention to them in another. We postulate two hypotheses on how successful (i.e., disruptive) trolling varies according to context: that trolls’ messaging strategies appear in different frequencies in political and interest forums (H1), and that context-matching strategies also produce longer futile conversations (H2). Using Hardaker’s categorization of trolling strategies on a covert–overt continuum, our statistical analysis on a dataset of 49 online conversations verified H1: in political forums covert strategies were more common than overt ones; in interest forums the opposite was the case. Regarding H2 our results were inconclusive. However, the results motivate further research on this phenomenon with larger datasets.

## Introduction

Online discussion platforms, such as online forums and news articles’ comment sections, connect millions of people daily. There are platforms and topics for everyone, hosting discussions ranging from seeking advice for personal trouble to heated debates on political matters. Many discussion platforms are vulnerable to malicious and disruptive behavior, which wreaks havoc in conversations and causes emotional distress to the people involved. Although online trolling is a diverse phenomenon, and perceptions towards it vary [9, pp. 65–89], the consensus is that it is ubiquitous and mainly disruptive, particularly because of the recent developments in using trolls to amplify polarization and political agendas, as well as to disrupt unwanted conversations and to spread disinformation [1, 5].

Considering the widespread agreement that Internet trolling can cause significant societal harm, it is surprising how little is known about the conversational strategies that trolls use. Evidence suggests, though, that trolling may manifest differently across contexts [9, 25]. Therefore, the trolling strategies used commonly in interest-oriented discussion forums may differ from the ones used in political debates. Most effective trolls may even be able to adapt their trolling strategies when they switch from one forum or discussion topic to another. Being aware of such differences in trolling strategies would be important in order to combat the ways by which trolls destroy civic conversations.

This paper’s findings come from a research project that has been launched to address the problem of trolling. Under the course of our research, we have made an initial observation that trolls seem to use different trolling strategies in political and interest discussions. Using a small dataset of 68 online discussions around political or societal themes (climate change, Brexit) and interest themes (cats, fitness), all of which included successful (i.e., response-inducing) trolling, we tested two hypotheses: that successful trolling strategies would indeed be applied with different frequencies depending on the topic of discussion (H1), and that the reply chains to trolls would also differ in their length, depending on the strategy used by the troll (H2). For distinguishing different trolling activities, we utilized the already well-established categorization by Hardaker [15] that describes six different trolling strategies along a covert–overt continuum.

The amount of data is so far limited, but our analysis suggest that H1 holds. We found a statistically significant difference between successful trolling strategies in political vs. interest discussions: in political discussions trolls apply covert strategies (i.e., subtle and non-apparent) more often than in interest discussions, where the strategies contrariwise are predominantly overt (i.e., noticeable and direct). On the other hand, we could not confirm H2 about reply chain lengths. The limited amount of data, however, pointed towards the direction predicted by the hypothesis: that covert trolling would lead to longer derailed discussions in political discussions, while overt strategies would do the same for interest discussion. The lack of confirmation to H2 notwithstanding, our findings have both academic and real-life implications, which we will cover in the Discussion.

## Theory

Our hypotheses did not result from serendipitous discoveries but had a theoretical backing that sensitized us to pay attention to their possible existence.

Trolls take advantage of the ambiguities of computer-mediated communication and the vulnerabilities of internet discussion communities to lure others into fruitless, frustrating or circular discussions and to waste their time [16]. Trolling involves a process of learning the social practices of a community, assimilating to them, and then violating these practices to create disruption [8, 25]. Trolling behaviors and perceptions of trolling are context-bound: they differ according to platform and community [9, 16, 25]. The motivations for trolling are similarly heterogeneous, including both amusement and political influence [3, 16]. Therefore, also the most common strategies used to successfully troll other participants on a discussion forum are context-dependent.

Previous studies have illustrated various types of trolling. They have often oriented to analyzing and understanding one type of trolling at a time, such as memorial page trolling [22], signalling of in-group/out-group membership [11], LOL trolling [17], and political trolling [1, 9]. In more generalizing depictions, differences between trolling styles have been illustrated e.g. by distinguishing between light or humorous trolling vs. (malevolent) serious trolling or ideological trolling [9, 10]. Community norms [19], platform, conversational style, motivations, and enabling factors all have an effect on the differences in trolling behaviors, as well as how they are interpreted by community members [9]. Therefore, considering the context-bound nature of trolling, it makes sense to study how trolling strategies vary according to context, and whether trolls behave differently in light conversations as opposed to more serious political conversations. While many of the above-listed studies have not presented typologies of different trolling strategies or styles, Hardaker’s [15] categorization of six comparable categories (Table 1) does that, and places different strategies onto a continuum ranging from covert trolling strategies to more overt ones. In our study, we adopt this categorization to classify our data, and to analyze the differences in trolling styles on political and interest forums.

**Table 1.** Hardaker’s [15] six trolling strategies on a covert-overt continuum

Strategy type	Strategy	Definition
Covert	Digression	Luring others into off-topic discussions by spamming, partaking in cascades or introducing tangential topics (e.g., as in [16]).
	(Hypo)criticism	Excessive criticism of others, e.g. on their punctuation while possibly committing the same errors oneself.
	Antipathy	Creation of a sensitive or antagonistic context through purposeful provocation, in order to manipulate others to produce emotional responses.
	Endangering	Giving out poor advice under an innocent guise, and others are compelled to respond in order to protect others.
	Shocking	Posting about taboos or sensitive subjects, such as religion, death or human rights.
Overt	Aggression	Deliberate and open aggressing of others into retaliating (e.g., by name-calling or foul language).

### Hypothesis 1: The Frequencies of Trolling Strategies Are Different in Political and Interest Forums

The relevance of a comment in an online forum depends on the content that has started the conversation. For example, a discussion in an online newspaper’s comment section happens in the context of the related news article. Similarly, in Reddit (a popular online news aggregator and discussion forum) a message is visible in relation to a “subreddit” (a discussion section) and an original post within it. Therefore the boundaries for the discussions that unfold are set to a specific topic that also sets the conversational context [18, 20]. This affects the expectations people have about the discussion and its style, and thus they tend to accommodate their posts to this context [29].

*Relevance theory* [26], which builds on Gricean maxims [12, 13], may help to illustrate why some posts on these forums manage to attract people’s attention far better than others. A post’s relevance is determined by not only its relevance to the assigned topic and the on-going conversation, but also its understandability. Relevance theory states that human cognitive mechanisms have a universal tendency of selecting most potentially relevant stimuli out of a variety, and to maximize the relevance of processed inputs, therein using the available processing resources most efficiently [26, Ch. 3.1–2]. The cognitive principle of relevance deems some messages more appealing or understandable than others, also making them more relevant [26]. We argue that along with contextual norms assigned by the discussion topic, relevance also dictates the conversation’s flow – in particular what type of posts (and thus trolling strategies) are deemed more relevant, and which posts incite more subthreads.

Compared to other less serious arenas, political forums discussing larger societal issues orientate more strongly toward more serious deliberative discourse or debate, and exhibit higher levels of interactivity and topical coherence [28] . They are to some extent similar to content-based and knowledge-based discussions on social media [18], and show less off-topic posts, as users’ contributions to the discussions are more likely to address previous posts in a manner befitting a real debate [28, pp. 15–17]. News discussion is largely opinion-based, and so participants also expect to be communicating with people coming from varying or opposing viewpoints [18, 27]. Thus, the general style of political forum discussion is different compared to interest topics. *Consequently, we believe that political forum discussions are more vulnerable to covert trolling attempts by being more neutral, information-centered and less personal.*

Contrarily to political arenas, interest forums serve as spaces for bonding with people with similar interests, beliefs or hobbies [4, 21]. Central motivations for joining these communities include information exchange, social support, and most of all friendship [24]. Essential for many such groups is creating an environment of camaraderie and supportive solidarity to enhance fun and a sense of belonging, which is why insults are taboo and confrontation minimized [4]. In general, interest forums invite contemplation on personal experiences, friendly exchange of feelings and anecdotes, and supportive information-sharing about the hobby or interest with other enthusiasts [4, 14, 20, 24]. We argue that due to the high relevancy of posts containing friendly support or personal experiences in this context, posts violating its taboos (e.g., insulting others) are also more cognitively relevant. This is because resolving and condemning such posts contributes to maintaining the key elements of the forum, such as a safe and friendly environment. Of course, conversations on online newspapers’ comment sections under interest-related articles do not necessarily form even a loose community. However, we consider it likely that these conversational arenas maintain some similar functional features as more close-knit communities like r/cats on Reddit. This is why *we maintain that interest forums match with overt strategies*, i.e. they are more vulnerable to more personal and visible overt trolling attempts like direct insults. Therefore, in summary, we hypothesize that:

- **H1:** The frequencies of covert and overt trolling strategies are different in political and interest forums.

In particular, we hypothesize that covert trolling is common in political discussion while overt trolling is common in interest forums.

### Hypothesis 2: Trolls Can Derail Others into Longer Futile Discussions by Choosing Trolling Strategies According to the Type of the Forum

Our second hypothesis is derived from the first one. If trolls match their trolling strategy to the type of the online forum, this may be because they know (consciously or sub-consciously) it will be more effective. One method for measuring the effectiveness of trolling is to measure the amount of engagement that a message manages to garner from others in the discussion.

Along with relevance theory, the *theory of common grounding* [6, 7] provides a theoretical justification for why trolls succeed in capturing other people into long unfruitful discussions. In well-intended communication, conversational parties engage in common grounding – a ‘collective process by which the participants try to reach a mutual belief that they have understood what each other meant’ [6, p. 223]. Following the premises of this psycholinguistics-derived theory, all contributions to a conversation need to be grounded, i.e. turned into mutual knowledge, by providing evidence that the message has been understood [6, 7]. All participants in the conversation are also expected to engage in resolving breakdowns in the case of possible misunderstandings. An unintelligible action thus calls for an explanation from its performer. This requirement for providing an explanation, in turn, is highly amenable for exploitation if one wishes to act as a troll. By resisting the norms of common grounding and accountability, a troll can prolong the time their posts attract attention.

As mentioned, contextual differences require learning the conversational conventions of a given online forum in order to gain access to the type of interaction others on the forum usually deem relevant [8, 9, 26]. Similarly, we state that relevant posts are seen as worth the collaborative efforts of grounding in case of breakdowns; in an asynchronous discussion space with a multitude of overlapping posts only discussion-relevant breakdowns are attended to. Consequently, we argue that participants on political forums are more prone to engaging in long grounding efforts when the conversation breaks down due to issues matching with the functions of the discussion space: misunderstandings or view point differences in informational content or correctness. On the other hand, we claim that people on interest forums are more inclined to engage in long conversations on personal experiences and issues related to the individual participant, which is why more collaborative effort will be expended on resolving the matching overt trolling attempts like unintelligible actions or attacks against a participant’s person. Therefore, our hypothesis H2 is, as already stated in the section’s title:

- **H2:** The quantity of replies to trolls will vary in different types of forums depending on the employed trolling strategy.

In particular, covert strategies would incite longer conversations on political forums, whereas overt strategies would have the same effect on interest forums.

## Data

Through selective sampling of online forums, we have manually acquired a corpus of conversations containing trolling. Keeping in mind our two hypotheses, we have selected several differing platforms to increase the heterogeneity of conversational and trolling styles. The corpus covers several discussion areas on *Reddit* and comment sections on English language online newspapers, including *the Telegraph*, *the Guardian* and *the Washington Post*. Having a large readership, these are influential media platforms that are likely to be targeted by trolls.

Considering our interest in both political and interest online discussions (see Sect. 2), our corpus includes two kinds of conversation topics: one around political issues (*climate change* and *Brexit*) and the other around interest discussions (*cats* and *fitness*). Important political topics, especially climate issues and Brexit , are likely to attract serious or ideological trolls wishing to disrupt or polarize the dialogue (e.g., [2, 3, 23]) Interest topics, in turn, such as apolitical and more everyday hobby-related discussions, may be vulnerable to “light” trolls if the topic is dear to the community (e.g., horses [14] or soap operas [4]).

In this data collection process, we have continued browsing the above-listed forums and their topic-specific discussion spaces until we have identified 2–5 conversation threads for each topic on each platform. We have particularly looked for activity-rich discussions in order to find successful trolling that has managed to elicit a lot of responses. Here successful trolling has referred to managing to formulate posts and/or responses to others’ posts that provoke others into responding directly or indirectly. Comments like ‘Don’t answer him, he’s a troll.’ and troll-triggered off-topic arguments among other participants have also qualified as responses. For the online newspaper comment sections, successful trolling has typically meant 8–15 response posts in a thread triggered by the troll, while on Reddit the range has been 15–20 replies. The differing numbers are due to the average number of replies having been smaller in newspaper comment sections as compared to Reddit, and the need for context-sensitivity as some topics inspired more replies in general than others, even within the same platform.

Finally, we have tagged all the trolling content in this dataset following Hardaker’s [15] six-category typology (see Table 1) where the trolling strategies can be located on an covert–overt continuum. We have used both conversationalist and researcher intuition to recognize what would have qualified as trolling in Hardaker’s study, labeling instances of trolling according to her categorization to gain a comprehensive dataset [14, 15].

## Results

Most trolling styles in Hardaker [15] could be found in each of the selected topics, with Brexit and climate change on the political axis, and fitness and cats on the interest axis. Table 2 presents examples.

**Table 2.** Examples of trolling using different strategies.

Strategy	Example start of discussion
Digression	*Political (climate change)*:
	Makes me wonder what flat earthers think since the flat earth is surrounded by ice walls.
	*– AccelHunter, Reddit, April 2019*
Hypocriticism	*Political (Brexit):*
	@Peter Wayde
	Peter, if you can’t even punctuate a sentence “why should we take notice you?”
	(heavy sarcasm)
	PS, “the causes will be the causes” is terrible syntax.
	*– Charles Hinton, the Telegraph, 16 May 2019*
Antipathy	*Political (climate change)*:
	It’s comments like this that make me realize how ignorant the Western left really is
	To you, the two sides are “the side I agree with personally” and “the side that is inherently wrong and evil”. There’s no middle ground. Everything is black and white and that’s that.
	*– Dreamcast3, Reddit, May 2019*
Endangering	*Interest (fitness)*:
	Im forced to take steroids to keep lifting
	Nothing will help my knees pain, been living with this life breaking pain for 10+ years, if i want to keep doing what i love, i have to take steroids.
	*– postashio, Reddit, June 2017*
Shocking	*Interest (cats)*:
	Let people have cats but just remove the cats claws and teeth.
	*– Viking76, the Telegraph, 12 June 2019*
Aggression	*Interest (cats)*:
	Why are cat owners less happy, you ask?
	Many cat owners are angry, man-hating, feminist spinsters - who cannot be happy.
	*– Yankees_Fan, the Washington Post, 5 April 2019*

### Are the Frequencies of Covert and Overt Trolling Strategies Different in Political and Interest Forums (H1)?

Our first hypothesis (H1), more specifically, was that trolls would be more likely to use covert trolling strategies (digression, (hypo)criticism or antipathy) in political discussions and overt strategies (endangering, shocking or aggression) in interest forums. To evaluate this hypothesis, we counted the frequency of each trolling strategy used in each discussion in our sample. We created two larger groups of trolling (covert and overt) by pooling together the frequencies of the three first and the three last strategies. This resulted in a $2\times 2$ frequency matrix whose values are presented in the sub-totals in Table  3.

In the preparation of this table, we removed the following cases that would have confounded our analysis. First, 13 discussions could be classified both as covert and overt trolling. After their removal, each discussion represented exclusively either covert or overt trolling. Second, there were 4 trolls (identified by their nickname) that appeared several times in our data (in 9 discussions altogether). To remove the possibility that their behaviors would be over-represented and would thus skew our data, we used a random number generator to sample only one discussion from each troll in our analysis. In one case, both confoundments were present within the same discussion. As a result, altogether we removed 19 discussions from the analysis. Table  3’s content is what remained after these preparations.

**Table 3.**
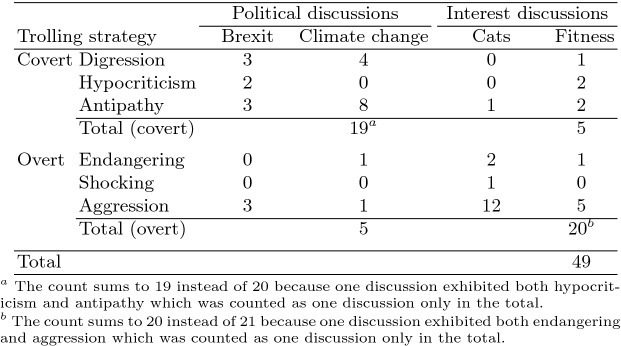
Examples of trolling using different strategies.

Already with a plain visual inspection of the frequencies, our hypothesis seemed to be true: there were more discussions in the political-covert quadrant than in the political-overt quadrant (19 vs. 5), and the inverse held in the interest-covert and interest-overt (5 vs. 20) quadrants. We confirmed the hypothesis by comparing frequencies between categories using a *Chi*-square contingency table analysis: in political discussions, covert trolling was more frequent while the opposite was true for interest discussions ($p < .0001$). Thus *H1 was confirmed*: trolls appear to use more covert trolling styles to (successfully) disrupt political conversations, whereas for invading interest conversations they use more overt styles.

We also studied how the removal of the fore-mentioned 13 discussions (where trolls had applied both overt and covert trolling strategies) had possibly skewed our findings. We included the removed discussions in our analysis by assigning them either to an overt or covert category. We implemented the assignment so that the frequencies between the categories would come as close to each other as possible, thus making it maximally difficult to find differences in a statistical test. Out of the 13 discussions 8 were political, 1 of which included a troll who had also appeared in another discussion in our data. We assigned the resulting 7 discussions to the overt trolling category, resulting in a 19 vs. 12 comparison between covert and overt strategies in political discussions (instead of 19 vs. 5; see Table  3). The remaining 5 discussions that had been removed were interest-based discussions, where covert strategies had been rare. We assigned all the 5 discussions to the covert group, thus yielding a 10 vs. 20 comparison (instead of the earlier 5 vs. 20). We finally repeated our test for frequency differences, and again found a statistically significant difference ($p<.05$), thus further confirming H1.

A closer look at Table  3 suggests that covert digression and antipathy strategies were particularly common in politically oriented discussions. Aggress trolling was also found in some cases (see Table  3), but the proportional amount of aggress trolling behavior was smaller than in interest conversations. In interest topics, in turn, successful trolls seemed to commonly exploit overt aggress and endanger strategies, attacking others directly or feigning concern about endangering issues like steroid use. It must be noted that in fitness discussions the difference between covert and overt strategies was very small, arguably because trolling instances were harder to find. With a larger dataset the above-stated possibilities may be studied further.

### Can Trolls Derail Others into Longer Futile Discussions Choosing Trolling Strategies According to the Type of the Forum (H2)?

As a follow-up for hypothesis H1, we specifically predicted in hypothesis H2 that the matching pairs of trolling strategy and discussion type (i.e., covert–political, overt–interest) would not only be more frequent but also, from the troll’s point of view, more “successful” in luring others into longer arguments. The success could be measured by the number of replies that others would post to the troll’s messages. Long chains of replies would best serve the trolls’ interest of creating havoc and destroying civic discussion in online spaces. The length of individual posts was not considered due to the fact that it may vary in online discussions for several reasons which cannot be controlled here.

To evaluate hypothesis H2, we counted the number of replies that others had posted to the discussion thread after the trolls’ original message. If the trolls themselves engaged in these subsequent discussions, we excluded their messages from these counts. We then compared the lengths of the reply chains in the $2 \times 2$ quadrants consisting of covert vs. over trolling and political vs. interest discussions. For this comparison, we used ANOVA, which is a method suited for analyzing differences between scalar values between categories.

Table 4 presents the data used in the analysis. Similarly with H1, also here a visual inspection suggests that the hypothesis could indeed hold: the covert-political and overt-interest matches have longer reply chains than the other pairs. However, this time we could not confirm this impression statistically: in a one-way ANOVA on political discussions, covert trolling did not lead to longer chains than overt trolling ($p = .279$). In the same analysis on interest discussions, overt trolling did not lead to longer chains than covert trolling ($p = .284$). We also carried out a two-way ANOVA with the strategy type (covert/overt) and the theme (political/interest) as factors, with an interest in the test’s interaction term that could test if the length variable’s relationship is inverted when analyzing the two different discussion topics. The interaction term was closer to a statistical significance, but not sufficient for any conclusions ($p = .129$). Correcting the length variable distributions’ skewness by square root transformation, or using non-parametric *U* tests did not yield significant results either. Thus, *H2 was not confirmed*.

**Table 4.** Lengths and standard deviations of the reply chains to troll’s posts.

General trolling strategy	Average reply chain length	
	Political discussions	Interest discussions
Covert	15.6 $(sd = 11.5)$	10.8 $(sd = 5.2)$
Overt	9.8 $(sd = 2.0)$	18.4 $(sd = 15.1)^\text {a}$

$^\text {a}$ One discussion was excluded due to an excessive number of replies (590).

The reason for this failure becomes apparent when one inspects the numbers of cases in each quadrant. The earlier-presented Table  3 shows that the data contained only 5 cases of mismatching strategy–discussion pairs (i.e., political–overt and interest–covert). Statistically significant findings were not attainable with such a small dataset size.

## Discussion

To recap, our first hypothesis was that commonly used successful trolling strategies differ according to the conversational context of the forum: political–covert or interest–overt. It was validated by a *Chi*-square analysis, which encourages further studies on the phenomenon with larger datasets. The second hypothesis was that covert strategies produce longer futile conversations in political arenas, whereas overt strategies drag on longer arguments in interest conversations. This claim was not supported by our statistical analyses at this point, but the data suggest it plausible for larger datasets to yield better results.

A better dataset would include a larger number or conversations, ranging through a greater variety of topics on the political and interest axes, including also unsuccessful troll posts. It would also allow for a more specific analysis of different trolling strategies, like the ones that Hardaker [15] identified. Our data is, of course, insufficient at the moment due to its size and the limitations of sampling trolling based on conversation-inherent dynamics. For the moment, classification into a category of trolling strategies per Hardaker [15, p. 68] requires several posts from the troll to determine whether the poster could be trolling others. This requirement means that our analysis addresses only successful trolling attempts where even the smallest attempt has led to a desired effect (from the troll’s point of view). Sampling and analyzing also unsuccessful trolling is a problem to be resolved in future research, and will allow more conclusive findings.

We also have other considerations that future research needs to address. First, how exactly the nature of the conversational space and its norms (as theorized by Kirman et al. [19]) affects communicational breakdowns. Now, the results of this study already implicate that transgression of contextual norms involves using a matching trolling strategy: trolls create posts that have high cognitive relevance in the discussion space. They also show that trolling style is not bound to individual and unique situations only; there are more general patterns in trolling that transcend forum and topic boundaries (e.g. Brexit), and certain types of forums can be expected to be vulnerable to matching trolling strategies. In political discussions, this means assimilating to the fact-based style, seeming (superficially) well-informed and topically coherent, citing (pseudo-)scientific sources and referring to field specific terminology, while baiting others for instance with antagonistic interpretations of related information, epistemological controversy or incoherence. In contrast, the interest context seems to give focus to trolling that attacks the friendly and supportive discussion’s main functions: here successful trolls do not require fact-based or topic-related expertise, high topical coherence or objectivity, but can instead overtly violate contextual boundaries by striking an emotional chord within the community. Thus, in the constant and multi-sided flow of posts with different and possibly overlapping agendas, the cognitive principle of relevance seems to dictate that posts matching with the functions of the discussion space gain most attention and manage to launch further discussions. The relatedness of more general contextual features and (successful) trolling strategies needs to be addressed more carefully in further research.

This also gives rise to further considerations beyond those that we put forward in our hypotheses. In particular, we find it worthwhile to consider relevance theory more broadly in the context of analyzing trolling. A relevance theoretical approach helps to further explicate the relationship between trolling and expectations of context-specific posts. Firstly, why some troll posts are noticed in the discussion while others receive very little attention, and secondly, why people engage in selected communicational breakdowns, despite their redundancy, provocativeness and frustrating effects. In interest discussions, for instance, participants seem to pay attention to overt troll posts because they seek to resolve norm-violations in order to reach common grounding and to maintain the friendly atmosphere. Arguably, participants on political forums put emphasis on factual correctness and enjoy sharing knowledge, which is why they are more inclined to be baited by epistemic incoherence or challenges against information they have provided. Thus, another possible course for future studies could involve deepening our understanding on how exactly discussion spaces give higher cognitive relevance to certain trolling strategies than others, e.g. why exactly certain posts are relevant to the people partaking in given discussions.

An issue to be aware of is that the results of the research presented in this paper, and in more extensive studies in the future, might be used for malicious purposes by aspiring trolls and bodies who are interested in large-scale misinformation campaigns. However, we believe that the results we presented here are mostly known to trolls already, whereas other discussants on online forums are probably less informed about trolling strategies. This makes them more vulnerable, which is why the results should yield positive results in raising awareness.

Assuming that the finding from H1 survives the test with a larger dataset, and H2 can eventually be proved, the implications are that we can expect certain types of online forums to be vulnerable to specific types of trolling strategies. The findings of this study already take us a step closer to identifying a given forum’s weak spots that enable trolling behaviors, thus helping in predicting and detecting trolling attempts. Developing awareness of the type of lures trolls use to attack different conversational groups would arguably also improve conversants’ resistance to trolls’ harassment. Future studies with larger sets of data will likely enhance the opportunities for identifying trolling patterns out of larger collections of online conversations, and therefore take us closer to more accurate automatizations of trolling detection and prevention, and moderation practices. Considering the recent developments in organized trolling of political discussions, detecting trolling patterns in these arenas on a larger scale would help in battling trolling used in information operations and to ensure democratic public spaces for online civic discussion. On the other hand, this would also help in ensuring that minority groups, for instance, will have safe spaces for meeting others with similar experiences, not having to be terrorized by trolls who seek only to amuse themselves or to oppress others.
